# Roles and responsibilities of clinical ethics committees in priority setting

**DOI:** 10.1186/s12910-017-0226-5

**Published:** 2017-12-01

**Authors:** Morten Magelssen, Ingrid Miljeteig, Reidar Pedersen, Reidun Førde

**Affiliations:** 1 Centre for Medical Ethics, Institute of Health and Society, University of Oslo, Pb. 1130 Blindern, N-0318 Oslo, Norway; 2 Department of Research and Development, Haukeland University Hospital, Bergen, Norway; 3 Research Group in Global Health Priorities, Department of Global Public Health and Primary Care, University of Bergen, Bergen, Norway

**Keywords:** Clinical ethics committee, Priority setting, Rationing

## Abstract

**Background:**

Fair prioritization of healthcare resources has been on the agenda for decades, but resource allocation dilemmas in clinical practice remain challenging. Can clinical ethics committees (CECs) be of help? The aim of the study was to explore whether and how CECs handle priority setting dilemmas and contribute to raising awareness of fairness concerns.

**Method:**

Descriptions of activities involving priority setting in annual reports from Norwegian CECs (2003-2015) were studied and categorized through qualitative content analysis.

**Results:**

Three hundred thirty-nine reports from 38 CECs were studied. We found 78 activities where resource use or priority setting were explicitly highlighted as main topics. Of these, 29 were seminars or other educational activities, 21 were deliberations on individual patient cases, whereas 28 were discussions of principled or general cases. Individual patient cases concerned various distributional dilemmas where values were at stake. Six main topics and seven roles for the CEC were identified. CECs handle issues concerning the introduction of new costly drugs, extraordinarily costly established treatment, the application of priority setting criteria, resource use for vulnerable groups, resource constraints compromising practice, and futility of care. The CEC can act as an analyst, advisor, moderator, disseminator, facilitator, watch dog, and guardian of values and laws.

**Discussion:**

In order to fulfil their responsibilities in handling priority setting cases, CECs need knowledge of both the ethics and the institutionalized systems of priority setting. There is potential for developing this aspect of the CECs’ work further.

**Conclusions:**

The Norwegian CECs are involved in priority setting decisions where they can play multiple constructive roles. In particular, they advise and raise awareness of ethical aspects in resource allocations; bridge clinical practice with higher-level decisions; and promote fair resource allocation and stakeholder rights and interests.

## Background

Although the most influential priority setting decisions are made at the political level when healthcare budgets are allocated, priority setting takes place at all levels of the healthcare services. Priority setting can be implicit or explicit [1, 2]. Priority setting ranks services according to their importance and will therefore, by necessity, determine the distribution of services in a way that creates “winners” and “losers” [3]. Apparently, both politicians, hospital managers and clinicians are reluctant to be the ones who have to turn down patient requests for expensive healthcare services [4].

The moral impact of distribution decisions has for decades lead theorists to call for greater transparency and reasonableness when allocating insufficient healthcare resources [5]. The Scandinavian countries have been at the forefront of the international endeavour of systematic priority setting processes at a policy level [6, 7]. Even for affluent countries health expenditure rises in a way that threatens to overwhelm the publicly financed health system. In Norway, a set of priority setting criteria have been regulating policy and practice through specific laws and regulations. There have also been strategies to conduct legitimate and transparent priority setting processes at the macro level, such as the establishment of The National Council for Priority Setting in Health Care [8]. The 2016 white paper on priority setting in Norway presented a revised list of priority setting criteria (“utility”, “resource use”, and “severity”), and there was a substantial call for more involvement of stakeholders and stewards of the regulations in priority setting decisions at all levels of the health care system [9]. One of the specific actors the government called upon was the hospital CECs. According to the white paper, the CECs should contribute to the identification and open discussion of priority setting dilemmas at the clinical level.

In Norway, each health trust – comprising one or more hospitals and providing specialized (secondary and tertiary) healthcare – is required to have at least one CEC, of which there are currently 37 in total. Norwegian CECs have an advisory role only without any decision-making authority. Traditionally, CECs have discussed ethical challenges related to concrete clinical situations concerning one or more patients, held seminars for staff and contributed to the development of guidelines. Typical cases have concerned treatment-limiting decisions, communication and confidentiality, patient autonomy and the use of coercion [10]. However, lately the Norwegian CECs have also taken on more principled and general issues, so that issues of what can be broadly termed organizational ethics now comprise a sizeable proportion of cases [11, 12]. Internationally it is debated whether organizational ethics requires a different venue than the CEC as the latter’s traditional focus and primary competence is in clinical ethics [13]. However, the Norwegian experience is that the CEC, as a multiprofessional body with competence in law and ethics and a patient representative, can be well suited also for discussing organizational ethics, thereby helping to bridge the gap between clinicians and hospital management [11].

CECs are trained in analysing and discussing clinical-ethical dilemmas together with all stakeholders in a systematic way following a specific deliberation method [14] in order to promote openness about value judgments and justifications in daily clinical practice. CECs could therefore be thought to be helpful also in improving priority setting decisions and making these more explicit and transparent [15, 16]. Priority setting decisions involve weighing different interests, as do CEC deliberations on clinical-ethical dilemmas. We are unaware of any studies of how CECs are involved in priority setting issues at a hospital level and what roles they can have on the path to fairer distribution of health resources.

Since 2011 the Norwegian CEC system has been regulated through a national mandate issued by the Ministry of Health and Care Services.^1^ Importantly, according to the mandate one of the tasks is to “contribute to increased understanding of the relationship between clinical-ethical issues and questions related to resource management and priorities in the health trusts”. Through an inquiry to the European Clinical Ethics Network we learned that Norway is most likely the only European country where CECs have been mandated to work with priority setting specifically. This makes a study of relevant CEC activities in Norway particularly relevant. Our objective is therefore to assess *whether* and *how* the Norwegian CECs have worked with issues of priority setting and resource use, and *which issues* have been addressed.

## Methods

Qualitative content analysis was performed in accordance with common norms for such analysis [17]. Coding and categorization was informed by the researchers’ pre-formed understanding of the field of clinical priority setting and of CECs’ functions in other contexts. All available annual reports from the Norwegian CECs for the time period 2003-2015 – 339 in total – were assessed by two researchers (MM and RF) independently. The annual reports detail the CECs’ activities to some extent, yet do not provide identifying details about patients or health professionals involved in the clinical-ethical cases. All activities in which resource use, rationing or priority setting were explicitly mentioned as main concerns were registered and coded with regard to content. Tentative topics were then constructed inductively on the basis of the codes. The topics were discussed among the researchers and then revised.

For some cases thought to have been particularly illustrative, the original, anonymized case reports were obtained upon a request to the CEC. Together with the material from the annual reports, this content was coded according to which functions or roles the CECs were seen to exhibit when handling the cases described. Drawing on experience with CECs in other contexts yet mainly inductively from the present data, one researcher (IM) drafted the list and description of roles, which were then discussed among the researchers repeatedly, and revised. For the sake of giving the reader a sense of the CECs’ actual – and diverse – modes of operation some of the cases are presented in the Results section. These particular cases have been chosen because they concern a broad set of priority setting topics, illustrate the CEC’s different roles and were thought to be cases in which the CEC’s efforts made a difference for practice. In one sense, therefore, they are “success stories” and not necessarily representative for the entire set of cases in our material.

According to the Norwegian system, formal research ethics approval was not required for this work as the annual reports analyzed are publicly available material and as the case reports received from the CECs were anonymous. As the cases presented in the Results section have been very thoroughly anonymized we have deemed consent to publish from patients, next of kin and clinicians not to be required.

## Results

We found 78 activities where resources and/or priority setting were explicitly highlighted as main topics. Of these, 29 were seminars or other educational activities, 21 were deliberations on individual patient cases, whereas 28 were discussions of principled or general cases. In the annual reports, some activities were described extensively or in some detail, whereas others were only supplied as brief descriptions or even sometimes just as titles.

CEC activities were categorized in six (partially overlapping) topics. The topics and examples of each are presented in Table 1.

**Table 1:** Topics related to resource issues treated in seminars (S), individual patient case discussions (I), and principled/general case discussions (P)

Topics	Examples
Introduction of new costly drugs	New, costly drug for cystic fibrosis (P); co-payment for expensive drugs (I + P)
Extraordinarily costly established/existing treatment	Costly treatment for patient with rare condition (I); home ventilator treatment (I); costly drug for serious and rare psychiatric disease (I); requirements for patient conduct and compliance when treatment is particularly expensive (I)
Application of priority criteria	Age limits for lung transplantation (P); triage in the emergency department (P); Caesarean section “on demand” (S); priority setting in rehabilitation medicine (S); discussion of national priority setting criteria (S)
Resource use for potentially vulnerable groups	Illegal immigrant with serious chronic disease and repeated admissions (I); repeated cardiac surgery for substance abusers (P); priority setting when medical evidence is scarce (P)
Budget/resource constraints compromising good practice	The incompatible logics of “care” and “production” (S); reductions in staff and number of beds leading to poorer services (P); transfer of imminently dying patients to nursing homes (P); early discharge from hospital due to resources constraints (I)
Futility of care	Expensive life-prolonging treatment with questionable benefits (I); priority setting in intensive care (S)

Our analysis of the cases also led to the identification of seven roles and responsibilities assumed by the CECs. The roles and their potential impact are shown in Table 2.

**Table 2:** Roles and possible impact of CECs dealing with priority issues

Role	Potential impact	Exemplified by cases
Analyst	Clarify values/principles at stake and the impact of decisions	A, B, C, D, E
Advisor	Solve concrete dilemmas	A, C, D, E
Moderator	Contribute to fairer decision-making processes	A, B, C, D
Disseminator	Create awareness and disseminate knowledge among clinicians	A, C, E
Coordinator	Connect different levels of healthcare organizations	B, D, E
Watch dog	Recognize unfair prioritizations and alert relevant authorities	B, E
Guardian of values and laws	Ensure legitimacy and fairness in line with common values	D, E

Below, five example cases illustrating the CECs’ work and roles and some of the topics are presented in some detail.

### Extraordinarily costly treatment and futility of care: home ventilator treatment (case A)

A patient with a degenerative disease had been living at home dependent on ventilator treatment for years. The large team of nurses required was very costly for the municipality which footed the bill; thus, the municipal authorities wanted the patient to move into a nursing home. The patient and spouse resisted this, arguing that the patient’s quality of life depended heavily on being able to stay at home.

The CEC was asked if a patient could demand that care was provided at home instead of in a nursing home; the latter arrangement would entail significant savings. A team of CEC members went to the patient’s house to discuss, and the opinions of patient and spouse were noted. Later the CEC arranged a meeting where many stakeholders participated in a discussion on fair use of resources and the conflicting considerations of patient autonomy and justice in this case. Municipality representatives stressed how the resource use influenced other patients in need, with, for instance, a shortage of home-care nurses. The CEC followed a structured deliberation method for ensuring that the stakeholders were heard and relevant information was gathered. In a complementary report to all the involved stakeholders the CEC used a structured set-up and referred in detail to the information presented to the committee, the trade-off between values and principles, the national priority setting criteria, and the legal limitation of the patient’s right to claim treatment lacking substantial proven benefits. The committee advised the municipality to offer care in the nursing home only. The patient’s need for quality of care and improved communication was also acknowledged. The municipal health professionals and authorities found the meeting and report useful and reported that it made it easier for them to make a decision and abide by it.

### Extraordinarily costly treatment for a vulnerable group: access to experimental drugs for psychosis (case B)

A patient was severely affected by chronic psychosis of a subtype which does not respond to ordinary antipsychotic drugs, and was hospitalized. The condition, including the ability to communicate and cooperate, improved substantially when an experimental drug was tried. Due to this treatment, the patient went from a state of continuous suffering to a more acceptable quality of life. However, the drug was expensive, and the request for public financing was turned down by the Norwegian Health Economics Administration (HELFO).

The CEC discussed the case and recommended that public funds cover the costs because of the great burdens of the disease for the patient, for the family and for the healthcare system. In addition, the CEC argued that the costs of the drug should not be viewed in isolation from the significant savings; the patient’s improvement led to reduced need of care personnel and therefore reduced personnel expenses. Furthermore, as the patient group in question was small, total societal costs would remain low even if the decision to treat would create precedence. Chronic psychosis patients constitute a weak societal group; this also counts in favour of higher priority, according to the CEC. The CEC’s report was submitted alongside a successful appeal to HELFO.

### Priority setting and resource use for vulnerable groups: valve replacement surgery for intravenous drug users (case C)

Recently, a hospital had had several patients with intravenous drug use (IVDU) and endocarditis in need of a second cardiac valve replacement surgery. There were different opinions on whether this surgery should be prioritized as it is expensive, recurrence risk is high, and few manage to end their substance abuse. International and national guidance was lacking. The CEC invited relevant stakeholders and worked on the case for a long time. By using a model for systematic analysis, the committee made explicit many of those considerations decision-makers were confronted with when facing such choices. The discussion illustrated not only the importance of solid evidence for making a well-considered choice, but also the necessity of clarifying the values and principles that have an impact on priorities. Based on the principle of equal treatment and expected survival, the committee found that this group of patients should receive the same treatment as other patients for whom a corresponding treatment effect is expected. The majority of the committee members were of the opinion that this group should be offered surgery irrespective of whether the substance abuse was likely to persist after the surgery, giving less weight to the contested priority criterion of responsibility for own disease state. The discussion in the CEC lead to a change in hospital policy: before, all IVDU patients in need of second replacement surgery were declined surgery; now the decision is made on an individual basis.

### Introduction of new costly drugs: Immunotherapy and patient co-payment (case D)

The CEC was contacted by a clinician working with patients with metastatic lung cancer. A new drug based on immunotherapy had shown some effect for this group. Yet the drug was very expensive with yearly costs at €130,000, and the effect modest, providing 3 months increased survival on average. The national decision-making authorities (“Beslutningsforum”) had not concluded if the costs should be covered by the hospital budgets. Now, some patients wanted to buy the drug themselves and requested to have the drug administered by the hospital (as biweekly injections), and the doctor asked the CEC for advice in how to respond. Such a practice would be unprecedented in Norway’s publically funded healthcare system and hospitals. In this city at that time, no private clinics would offer to administer the treatment. Several stakeholders were consulted. The committee concluded that the principle of equal access is fundamental in the Norwegian health care system, and therefore it is not acceptable that only those who are able to pay should receive the drug as patients within a public hospital. The clinician was glad to have clearer arguments and support in the committee – and, subsequently, hospital management – when he had to turn down the patients’ requests. The patients were not satisfied, and some protested in the media. The CEC’s proposed policy, however, was recently reiterated in the government white paper on priority setting [9]. The committee found it necessary to bring the dilemma to a national level: when a drug has been proven effective and safe and a national-level evaluation of coverage is underway, what should be done in the meantime? There will be many similar situations in the future. The CEC thus referred the case for a discussion in the National council for priority setting. At the time of publishing the Council has commenced preliminary discussions on the case.

### Budget constraints compromising good practice: a “waiting list scandal” (case E)

A CEC at a large hospital discussed a case which in the media had been presented as a scandal. According to Norwegian regulations, patients on waiting lists for medical treatment must be given a deadline tailored to the individual for when treatment is to commence. If treatment has not begun on the specified date, the patient may complain. If treatment then is not commenced quickly, the patient is entitled to treatment at other healthcare trusts or abroad, with the original hospital footing the bill. In order to avoid excessive costs, some departments at the hospital in question prioritized expedite treatment for the patients who complained about missed deadlines. Those who did not complain, however, were left to wait longer, and the fear was that this group was, in effect, unfairly given low priority. General complaints about these practices from the staff to the management were not followed up.

The hospital’s CEO referred the case to the CEC and took part in the CEC’s discussion. The CEC stressed that most likely resourceful patients capable of advancing their rights and of complaining had here been advantaged, relative to the potentially less resourceful group who had not complained and who was thus unfairly disadvantaged. Among those who had not complained might have been the most seriously ill. The practice involved a breach with the principle of justice when the hospital let its own economic incentives and disincentives override the principle of equal treatment, the medical evaluation of need, and the assessment of severity, utility and cost-benefit ratios. However, the CEC pointed out that knowledge about the waiting list patients, and thus the actual consequences of the priority setting, was lacking. The CEC recommended that such knowledge was obtained, and that a system to identify patients whose deadline was approaching or whose medical condition was worsening while on the waiting list, was introduced.

### Other findings

The number of CEC activities involving priority setting and resource use increased throughout the period studied (13 years in total). The three most recent years (2013-15) saw 45% (35 of 78) of the CEC activities registered.

Findings also indicate that priority setting and resource use have been addressed more often by CECs in university hospitals than in medium-sized and smaller hospitals. Thirty-seven (47%) of the 78 activities were registered in university hospital CECs (comprising eight of the 37 CECs studied). Most of the discussions related to particularly resource-intensive treatment such as costly new drugs or intensive care were raised at university hospitals.

We were interested in whether CEC would sometimes express explicit criticism towards hospital management, and we found such criticism in five case reports (four principled/general cases, one individual patient case). Typically, management was criticised for underfunding or understaffing of important functions.

## Discussion

### Wide variation in topics

The results indicate that the Norwegian CECs have worked with issues of priority setting and resource use in a variety of ways, identifying such issues in individual patient cases and principled/general cases referred for CEC deliberations, and bringing such issues up for reflection and debate among health professionals in the hospital in seminars. As some of the examples illustrate, clinical-ethical cases of this sort might be both important and highly complex. The issues have appeared more often in recent years; two possible explanations for this can be, first, that awareness of priority setting issues in the healthcare services has increased; second, that recent years might have seen some new kinds of dilemmas, as in case D.

### The CEC as analyst, advisor and moderator

CEC members are trained in structured analysis of what is at stake in a clinical-ethical dilemma, emphasizing the values, moral principles and relevant law involved in the case. Dilemmas of priority setting and resource use ought to be no different than other clinical-ethical dilemmas in this regard, and the CEC can analyse and provide advice on such cases in light of priority setting criteria and relevant arguments from ethical debates in the field.

In addition, CECs emphasize process: as per the ideals of discourse ethics, CECs aim for stakeholder involvement in the deliberations, either directly (such as in case A) or by representative [18]. The CEC may thus provide the kind of structured, transparent and open deliberation process that some argue is an essential prerequisite for legitimate priority setting decisions [5].

The CEC might constitute a well-suited forum for priority setting discussions because contributing knowledge of ethics, law and medicine, and being broadly constituted with different professions and user representation. Ideally, then, the CEC may play a role in moderating more just priority decisions; arguably, cases A-E exemplify this.

### The CEC as disseminator

Several CECs arranged seminars on the 2014 governmental report on priority setting and the framework for priority setting suggested therein [19]. Others seminars concerned priority dilemmas affecting specific patient groups or medical specialities. In such seminars, participants are exposed to priority setting frameworks and their rationale, as well as given a forum for discussing implications for their own practice. Through seminars the CEC therefore can bring awareness of priority setting issues and knowledge of arguments and principles to a greater number of clinicians.

In case discussions, once it is agreed that a dilemma concerns priority setting, the dilemma can be analyzed within corresponding ethical frameworks and with relevant ethical terms and priority criteria. The CEC might therefore have an important role in identifying that the dilemma at hand is a priority setting dilemma, as is illustrated in all the example cases (A-E). As health professionals do not necessarily have other forums for discussing priority dilemmas, the CEC may play a significant role in ensuring that priority setting and resource use is discussed by clinicians. This may lead to increased awareness of dilemmas, competence (e.g., knowledge of priority setting criteria and moral arguments) and openness in clinical priority setting.

### The CEC as coordinator

Several of the cases (B, D, E) illustrate the CEC’s role as a coordinator or facilitator of communication between individual clinicians and clinical departments on the one hand, and hospital management and national institutions responsible for priority setting on the other. In these cases, the CEC helped identify and formulate the dilemmas and the values at stake before referring the case on. They thereby ensured that the dilemmas experienced at the clinical level were raised to a higher level. Case E also illustrates that concerns may not be taken sufficiently seriously in the hospital hierarchy when voiced by individual clinicians; the CEC, however, may be well positioned to highlight and speak up about problematic practices.

### The CEC as watch dog and guardian

The CEC can act as a stakeholder advocate – a “watch dog”. In case B, the CEC advocated for the patient’s perspective in the appeal process with the responsible office on the national level. In case A, the CEC turned out to support the concerns of the health professionals, underpinning these with arguments rooted in priority setting criteria. The CEC’s watch dog role can be important in promoting more just decisions; especially, arguably, in ensuring that patients’ right to healthcare is met. Cases B and C illustrate the CEC supporting groups that may be relatively neglected in the health services (here, patients with chronic psychosis and intravenous drug abuse, respectively). The CEC’s verdict and any advocacy, however, must be based on close weighing of the moral and medical arguments [20].

Relatedly, in our material there were also several examples of the CEC acting as a guardian of societal values. The CEC would sometimes interpret a case and a proposed line of action as presenting a challenge to commonly accepted values and moral principles. The CEC would then have a role in upholding such values, arguing that the courses of action that best protect these should be preferred. Case D is an example of this, where the principle of equality of access was highlighted and argued to be an overriding concern. The watch dog and guardian roles may also come into conflict, when, for instance, the health care needs of a patient or a patient group is pitted against tenets of professional ethics or priority setting guidelines.

There are some examples of CECs being willing to take potentially “unpopular” stances such as recommending against offering treatment for individual patients or patient groups; cases A and D are examples of this. In our findings, there are also some examples of the CEC criticizing departments or hospital management (e.g., case E).

### Are the CECs equipped to handle issues of priority setting?

In our estimation, cases A-E show the efforts of CECs being of some help in a set of priority setting dilemmas; evidently, then, CECs *can* be helpful in priority setting issues. However, our investigation was not suited to directly assess the quality of the CECs’ work with issues of prioritization. Thus, we cannot say whether the CECs actually were of help in the remainder of the cases identified in the study, or indeed whether they sometimes did more harm than good. The field of priority setting in healthcare is complex and requires the CEC to be familiar with priority setting criteria and specialized ethical debates, as several of the examples illustrate. Case B, for instance, saw the CEC weighing and adapting general principles (i.e., national priority setting criteria) to fit the complexities of an individual patient’s situation in a fair manner. Most likely such knowledge, some of which within the ambit of political philosophy, is not as widespread among CEC members as is knowledge of other aspects of clinical ethics.

There is a need, then, for the CEC to acquire specialized knowledge, for instance through tailored courses in the ethics of priority setting, or by recruiting at least one member with such knowledge. Seeing as how clinical-ethical cases appear more likely to occur at university hospitals, a case could be made that the most difficult clinical-ethical cases concerning priority setting should be handled mainly in university hospital CECs, at least at the present stage. Such CECs typically have professional ethicists among their members. However, it is not ideal to remove discussion from the hospitals in which the cases will have to be handled. It is also vital that the local stakeholders are able to participate in the CEC discussions. Furthermore, CECs in smaller hospitals have an equally important responsibility in inducing awareness of priority setting issues amongst local clinicians; this task cannot be transferred to university hospital CECs.

What authority should the CECs have in such cases? In Norway, as in many other countries, CECs have no decision-making authority. The CEC will analyse the case and provide advice if this is requested; it is then up to the clinician whether and how to use the CEC’s advice.

If a critic were to question the democratic legitimacy of a CEC’s handling of priority setting dilemmas then this would be part of our answer: the CEC does not make decisions; it only attempts to illuminate the case and provide advice. However, because only the most general priority setting decisions can be made at the legislative level, there is a need for agents “downstream” to carry on the responsibility of fair priority setting [21]. Increased transparency is called for in priority setting [5]; CECs promote transparency through presenting the relevant arguments in written case reports available to the stakeholders and sometimes to larger audiences. Arguably, therefore, a CEC deliberation, through being structured, transparent and open to diverse stakeholder perspectives, is able to confer not only moral but some political legitimacy on the conclusion reached.

### Strengths and limitations

The study’s strength is that it is a systematic investigation of the activities of all Norwegian CECs. The limitation is that some of the CEC yearly reports are brief and selective, without detailing topics and dilemmas, sometimes describing activities by title only. For this reason, it is likely that some relevant activities have been missed. In addition, the yearly reports and the case reports are the CEC’s own descriptions; possibly, the CEC’s roles and impact would appear in a different light if the experiences of the other stakeholders had also been consulted. From previous research and from our experience we contend that priority setting and resource use often figure as relevant considerations in CEC cases without necessarily being treated as the dominant or decisive issues [22]. Such issues, then, are likely to have been addressed in many more CEC cases than the ones identified in the study. One set of examples would be cases concerning the limitation of life-prolonging treatment for severely ill or dying patients, cases commonly addressed by CECs.

## Conclusion

Norwegian hospitals CECs play several potentially valuable roles in handling issues of priority setting and resource use in the health services. Clinical cases involving such issues may be both important and complex, and are likely to become more prevalent. In order to fulfil their responsibility, CECs thus need knowledge of both the ethics and the institutionalized systems of priority setting. There is potential for developing this aspect of the CECs’ work further. There should be openness about the CECs’ work, and their handling of cases should also be evaluated.

## Abbreviations

CEC: Clinical ethics committee
HELFO: Norwegian Health Economics Administration
IVDU: Intravenous drug use

## Footnotes

1 The national mandate is available at http://www.med.uio.no/helsam/english/research/projects/clinical-ethics-committees-in-hospitals/national-mandate-for-cecs.pdf
